# Protease cleavage of RNF20 facilitates coronavirus replication via stabilization of SREBP1

**DOI:** 10.1073/pnas.2107108118

**Published:** 2021-08-27

**Authors:** Shilei Zhang, Jingfeng Wang, Genhong Cheng

**Affiliations:** ^a^Department of Microbiology, Immunology, and Molecular Genetics, University of California, Los Angeles, CA 90095;; ^b^Institute of Systems Medicine, Chinese Academy of Medical Sciences & Peking Union Medical College, Beijing 100005, China;; ^c^Suzhou Institute of System Medicine, Suzhou 215123, China

**Keywords:** SARS-CoV-2, protease, RNF20, RNF40, SREBP1

## Abstract

COVID-19, caused by severe acute respiratory coronavirus 2 (SARS-CoV-2), has presented a serious risk to global public health. The viral main protease M^pro^ (also called 3Cl^pro^) encoded by NSP5 is an enzyme essential for viral replication. However, very few host proteins have been experimentally validated as targets of 3Clpro. Here, through bioinformatics analysis of 300 interferon stimulatory genes (ISGs) based on the prediction method NetCorona, we identify RNF20 (Ring Finger Protein 20) as a novel target of 3Clpro. We have also provided evidence that 3Clpro, but not the mutant 3Clpro^C145A^ without catalytic activity, cleaves RNF20 at a conserved Gln521 across species, which subsequently prevents SREBP1 from RNF20-mediated degradation and promotes SARS-CoV-2 replication. We show that RNA interference (RNAi)-mediated depletion of either RNF20 or RNF40 significantly enhances viral replication, indicating the antiviral role of RNF20/RNF40 complex against SARS-CoV-2. The involvement of SREBP1 in SARS-CoV-2 infection is evidenced by a decrease of viral replication in the cells with SREBP1 knockdown and inhibitor AM580. Taken together, our findings reveal RNF20 as a novel host target for SARS-CoV-2 main protease and indicate that 3Clpro inhibitors may treat COVID-19 through not only blocking viral polyprotein cleavage but also enhancing host antiviral response.

## Significance

SARS-CoV-2 has a main protease for viral polyprotein processing. But too few studies addressed this protease’s target of host factors. Here, we screen nearly 300 interferon-stimulated genes (ISGs) and identify that RNF20 is cleaved by 3Clpro, which prevents SREBP1 degradation mediated by RNF20/RNF40 complex, thus promoting SARS-CoV-2 replication. Our study will encourage people to develop drugs against not only coronavirus protease but also host factors.

COVID-19, caused by a novel coronavirus named severe acute respiratory syndrome coronavirus 2 (SARS-CoV-2) in December 2019, has devastated the globe as a pandemic that has infected more than 128 million people and caused more than 2 million deaths as of early April 2021 (1, 2). SARS-CoV-2 is an enveloped, positive-sense single-strand RNA virus containing a 30-kb genome, which has two cysteine proteases, papain-like protease (PLpro) and 3C-like protease (3Clpro), which are located in the nonstructural protein (nsp) regions nsp3 and nsp5, respectively, that mediate the functions required for viral replication and transcription (3, 4). In addition to the 11 cleavage sites in the viral polyproteins, the host proteolytic targets of SARS-CoV-2 3Clpro are still anonymous, while multifarious host proteins have been predicted but not yet validated experimentally as 3Clpro substrates.

The innate immune response initiated by pattern recognition receptors provides the first line of defense against invading viral infections. Pattern recognition frequently results in the production of type I interferons (IFNs), which further modulate the transcriptional induction of hundreds of IFN-stimulated genes (ISGs) to alert cells in an “antiviral state” that prevents virus infection and replication through diverse mechanisms (5, 6). The 3CLpro protease encoded by diverse coronaviruses including porcine epidemic diarrhea virus, porcine deltacoronavirus, and feline infectious peritonitis virus exhibited proteolytic activity on NF-κB essential modulator (7–9). However, it is not yet confirmed that the antiviral function of ISGs is also contracted by SARS-CoV-2 or its encoded proteins.

Here, we analyze ∼300 known ISGs and identify several ISGs that are vulnerable to SARS-CoV-2 3Clpro. Notably, E3 ligase RNF20 (also known as hBRE1A) was identified as the target of SARS-CoV-2 3Clpro for cleavage at Gln521, which impaired the function of RNF20/RNF40 (also known as hBRE1B), thereby hijacking sterol regulatory element binding protein 1 (SREBP1)-driven lipid metabolism for viral replication. These data uncover a mechanism acquired by SARS-CoV-2 to disarm host innate immune responses, and could be exploited for the development of antivirals to combat COVID-19.

## Results

### Identification of Host ISGs Targeted by SARS-CoV-2 3Clpro.

**Fig. 1.**
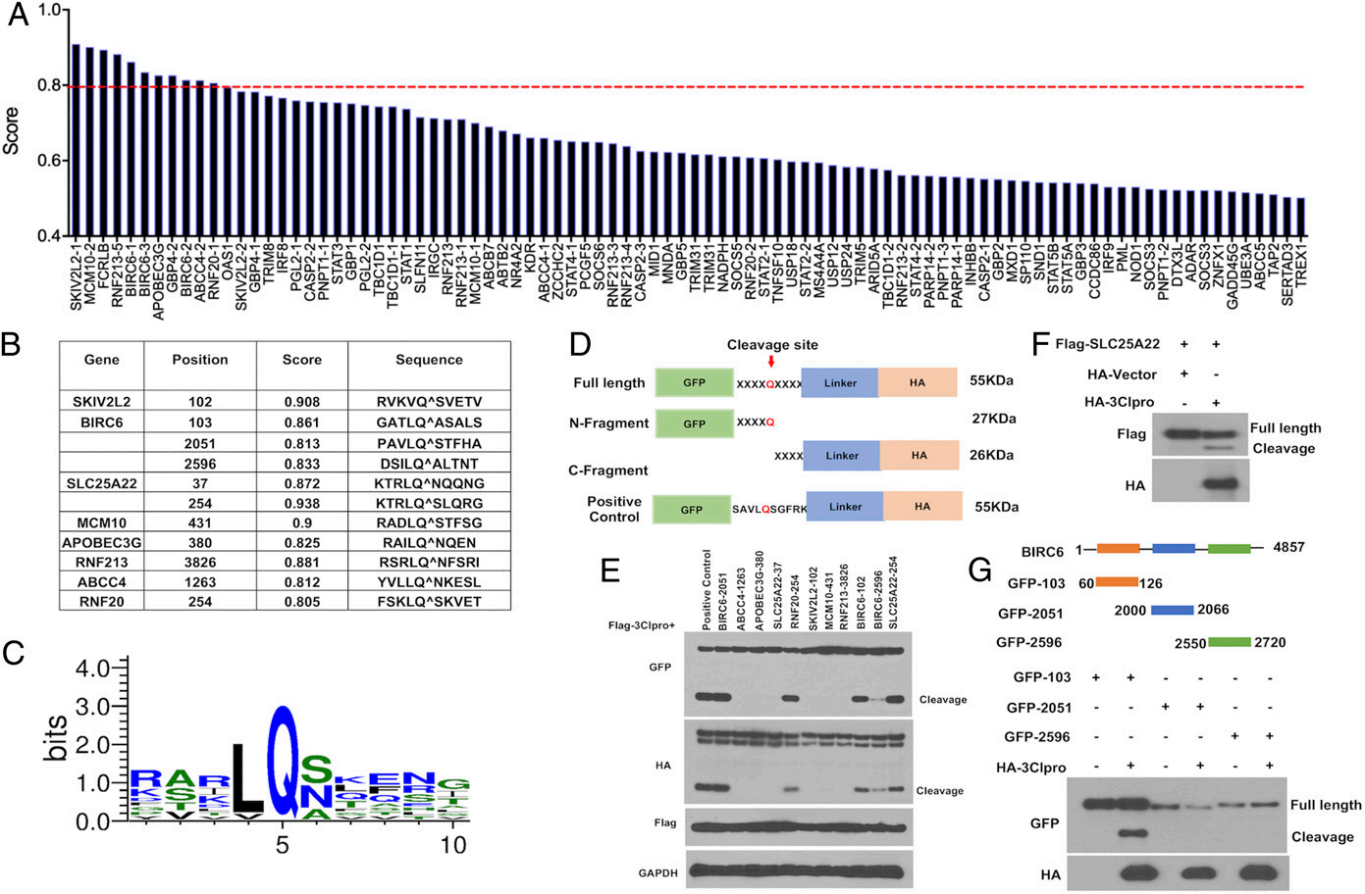
Identification of ISG targets by SARS-CoV-2 3Clpro. (*A*) Prediction of potential ISGs targets by SARS-CoV-2 3Clpro. The red line indicated the threshold value of 0.8. The candidate ISGs over the threshold were further explored. (*B*) The potential sequences and the position of the cleavage sites. (*C*) Logo analysis of the cleavage sites predicted from the potential ISGs cleavage sequences. (*D*) Diagram of the reporter construction with the predicted cleavage sequences. The predicted sequences listed in *B* were individually cloned into pEGFP vector, which would express GFP tag at the N terminal and Linker-HA tag at the C terminal of the cleavage sequence. The sequence SAVLQSGFRK was also cloned into the same vector, as a positive control. Upon cleavage, the full-length protein of 55 kDa will be processed into two fragments: N-GFP fragment (27 kDa) and C-HA fragment (26 kDa). (*E*) Immunoblotting analysis of the predicted sequences. The indicated expression vectors were transfected with Flag-3Clpro into HEK293T cells for 24 h. Cell lysates were subjected to immunoblot with the antibodies indicated in the figure. (*F*) Validation of SLC25A/22 cleavage. HEK293T cells were transfected with 2×FLAG-SLC25A22 and HA-3Clpro, and Western blot analysis was performed using the indicated antibodies. (*G*) Validation of BIRC6 cleavage. Three fragments of BIRC6 (60 aa to 126 aa, 2,000 aa to 2,066 aa, and 2,550 aa to 2,720 aa) containing the potential cleavage sequence at Q103, Q2051, or Q2596 were generated. HEK293T cells were transfected with the indicated fragments and HA-3Clpro for 24 h, followed by immunoblotting analysis with the indicated antibodies.

To investigate whether SARS-CoV-2 3Clpro can cleave ISGs to interfere with the host response, we applied the NetCorona 1.0 webserver to in silico analysis of 300 ISGs identified before by our laboratory (Fig. 1*A*) (10). NetCorona 1.0 is a web server that predicts coronavirus 3C-like proteinase (or protease) cleavage sites using artificial neural networks on amino acid sequences (11). To enhance the prediction credibility, we filtered ISGs based on scores exceeding a threshold of 0.8, and identified eight ISGs as potential targets of 3Clpro, including SKIV2L2, MCM10, APOBECG3, RNF213, and ABCC4, and RNF20 with one cleavage site, SLC25A22 with two cleavage sites, and BIRC6 with three sites (Fig. 1*B*). We further compared the cleavage site sequences of these ISGs candidates and found that SARS-CoV-2 3Clpro cleavage sites contain a conserved Gln residue at P1 position, and there are hydrophobic (Leu or Val) and aliphatic (Ser, Asn, or Ala) residues at P2 and P1′ positions, respectively (Fig. 1*C*).

To experimentally validate the predicted cleavage sites, we generated the reporter constructs with distinct cleavage target sequences of the selected ISGs fused with GFP (N terminal) and HA-tagged linker (C terminal) (Fig. 1*D*). Immunoblotting analysis with anti-GFP or anti-HA antibodies showed that all three predicted cleavage sequences of BIRC6 were vulnerable to 3Clpro, while only one sequence in each of SLC25A22 and RNF20 could be cleaved (Fig. 1*E*). The protein abundance of full-length Flag-SLC25A22 was reduced in HEK293T cells coexpressing 3Clpro compared with that coexpressing the control vector. This was also accompanied by the appearance of a faster-migrating band, presumably an SLC25A22 cleavage product (Fig. 1*F*). BIRC6 (baculoviral IAP repeat-containing 6, also known as Bruce/APOLLON) is a large IAP (inhibitor of apoptosis) with a molecular mass of 530 kDa. To further validate that 3Clpro could cleave BIRC6, GFP-tagged constructs harboring the indicated domains of BIRC6 containing predicted cleavage sites at Q103, Q2051, or Q2596 were generated (Fig. 1*G*). The results showed that GFP-103 was cleaved by 3Clpro, but no cleavage products were observed in GFP-2051 and GFP-2596, indicating that Q103 of BIRC6 would be the target site of SARS-CoV-2 3Clpro (Fig. 1*G*).

### The 3Clpro Cleaves RNF20 at Residue Q521 via Its Protease Activity.

**Fig. 2.**
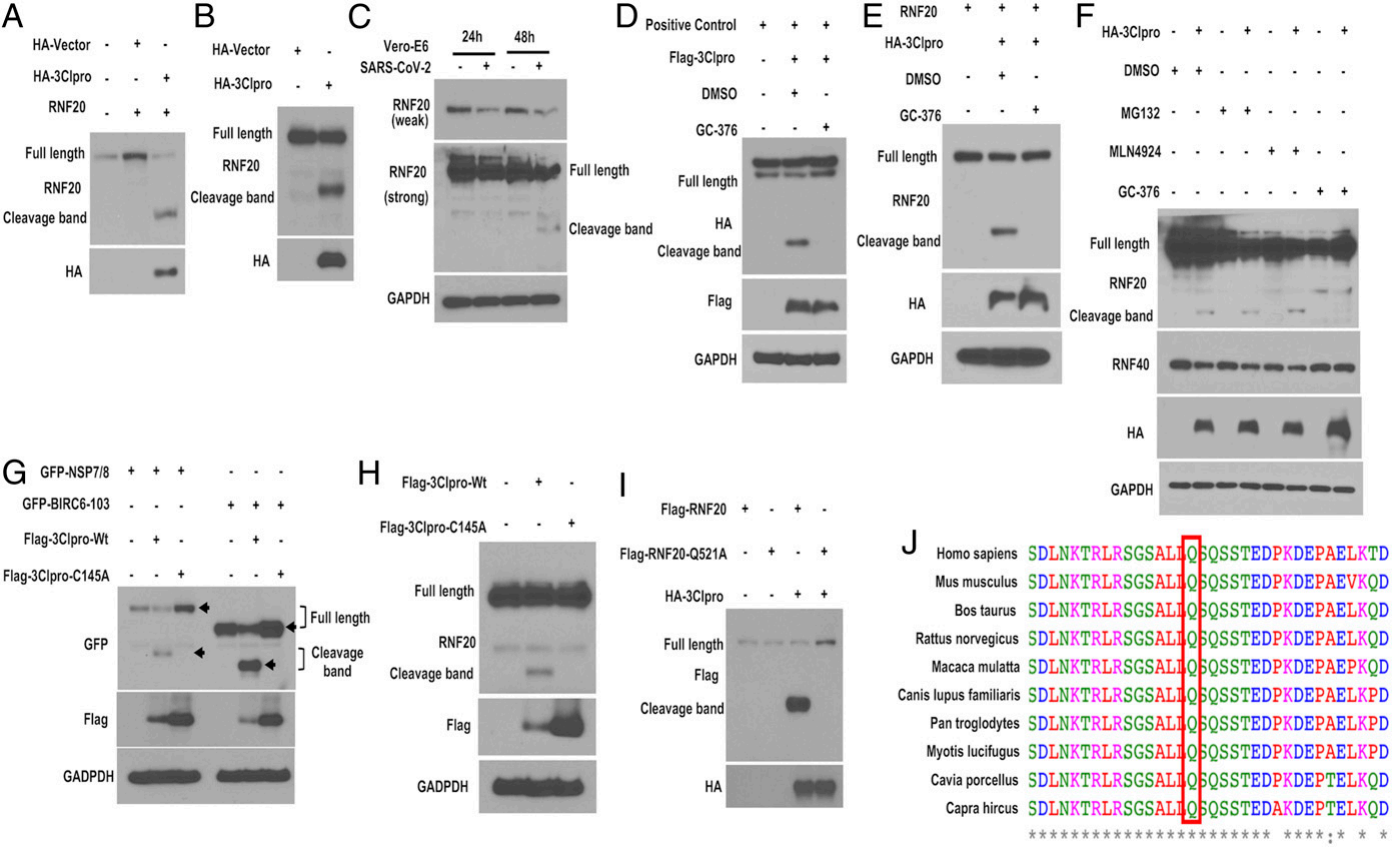
The 3Clpro cleaves RNF20 at residue Q521 via its protease activity. (*A*) Cleavage of ectopic RNF20 by 3Clpro. Vector expressing RNF20 (without Tag) was cotransfected with control vector or HA-3Clpro into HEK293T cells for 24 h. Cleavage of RNF20 was determined by Western blot using the specific antibody against the N terminus of RNF20. (*B*) Immunoblotting detection of endogenous RNF20 cleavage. HEK293 cells were transfected with control vector or HA-3Clpro for 24 h. Cell lysates were harvested for analyzing cleavage of endogenous RNF20 with the antibody against the N terminus of RNF20. (*C*) Endogenous RNF20 cleavage upon SARS-CoV-2. Vero E6 cells were infected with SARS-CoV-2 at MOI 2 for 24 and 48 h. Cell lysates were analyzed by Western blot to show the cleavage of endogenous RNF20. Glyceraldehyde-3-phosphate dehydrogenase was used as a loading control. (*D* and *E*) The positive control vector (carrying SAVLQSGFRK, also used in Fig. 1*E*) or RNF20 (without Tag) were transfected into HEK293T with HA-3Clpro for 20 h and then treated with either dimethyl sulfoxide (DMSO) or main protease inhibitor GC-376 (50 µm) for 12 h prior to measuring the cleavage of the indicated protein. (*F*) HEK293T cells were transfected with HA-tagged 3Clpro for 20 h, and then treated with DMSO, MG132 (10 µm), MLN4924 (1 µm) for 6 h or GC-376 (50 µm) for 12 h, followed by immunoblotting analysis with the indicated antibodies. (*G*) GFP7/8 and GFP-BIRC6-103 were cotransfected with wild-type 3Clpro or enzymatic dead mutant (C145A) 3Clpro into HEK293T cells for 24 h. Western blotting was performed to analyze the cleavage of the indicated protein. (*H*) HEK293T cells were transfected with wild-type 3Clpro or mutant C145A for 24 h and then subjected to immunoblotting analysis of endogenous RNF20 cleavage with the indicated antibodies. (*I*) HEK293T cells were cotransfected with a plasmid expressing FLAG-RNF20 or its mutant (Q521A) alone or in combination with a plasmid expressing HA-3Clpro for 24 h, and the cell lysates were analyzed by Western blotting. (*J*) Analysis of the protein sequences across species for RNF20 cleavage sites using Clustal Omega online service.

To experimentally determine whether RNF20 is the proteolytic target of 3Clpro, we transfected the expression vector encoding RNF20 with 3Clpro into HEK293T cells. Immunoblotting analysis using a specific antibody recognizing N terminus of RNF20 showed the generation of a faster-migrating fragment of RNF20 that was about 60 kDa shorter than the full-length RNF20 (Fig. 2*A*). To determine whether the endogenous RNF20 is also cleaved by 3Clpro, we further evaluated RNF20 protein expression in a 3Clpro-overexpressed HEK293T cell. As shown in Fig. 2*B*, an evident cleavage fragment of RNF20 was observed by using an RNF20-specific antibody. To confirm whether RNF20 cleavage under ectopic expression is relevant to SARS-CoV-2 biology, endogenous RNF20 was also evaluated in SARS-CoV-2−infected Vero-E6 cells. Interestingly, the protein levels of full-length RNF20 were decreased at both 24 and 48 hpi (hours postinfection) compared with mock-infected cells, while the cleavage fragment was observed at 48 hpi (Fig. 2*C*). Furthermore, we also found that all 3Clpro from different coronaviruses including SARS-CoV-1, SARS-CoV-2, and Middle East respiratory syndrome (MERS)-CoV were able to cleave RNF20 (*SI Appendix*, Fig. S1). This result indicates that RNF20 cleavage is a conserved mechanism among different CoVs.

GC-376 inhibits SARS-CoV-2 main protease through binding the catalytically active site of main protease (12). Consistently, GC-376 treatment could efficaciously inhibit 3Clpro-mediated cleavage of both SAVLQSGFRK and RNF20 (Fig. 2 *D* and *E*). Moreover, endogenous RNF20 cleavage by 3Clpro in HEK293T cell was also prevented by GC-376, but not by proteasome inhibitor MG132 and neddylation inhibitor MLN4924, indicating that RNF20 cleavage is independent of cellular proteasome degradation (Fig. 2*F*). To further validate that SARS-CoV-2 3Clpro-mediated RNF20 cleavage is associated with its protease activity, 3Clpro mutant encoding C145A was tested by cotransfection with GFP-BIRC6-103 or GFP-SARS-CoV-2-NSP7/8 which are located in viral replicase polypeptides harboring proteolytic sequence. As shown in Fig. 2*G*, compared to the wild-type 3Clpro, single amino acid changed at C145 abrogated cleavage of BIRC6 and NSP7/8, suggesting that C145 is essential for 3Clpro protease activity. Moreover, wild-type 3Clpro cleaved endogenous RNF20, whereas C145A mutant failed to do so, indicating that protease activity is indispensable for 3Clpro to cleave RNF20 (Fig. 2*H*).

In silico analysis in Fig. 1*B* shows that Q254 might be the cleavage site of RNF20. We calculated that the molecular weight of RNF20 from M1-Q254 was about 29 kDa, which cannot be seen in our immunoblotting detection, whereas the fragment of about 60 kDa was evidently identified by commercial antibody against 96 to 433 amino acids of RNF20, suggesting that Q254 was not the cleavage site. To identify the recognition site of RNF20 in 3Clpro-mediated cleavage, we further in silico analyzed the potential cleavage sequence of RNF20 in NetCorona 1.0 webserver regardless of the threshold, and identified Q521 as the candidate site which can produce a theoretical fragment (M1-Q521) with a molecular mass of 60 kDa. The immunoblotting analysis showed that RNF20 Q521A mutant was resistant to 3Clpro-mediated cleavage, as evidenced by the disappearance of the cleavage band between 55 kDa and 75kDa, while cleavage of wild-type RNF20 was not changed, indicating RNF20 is cleaved at Q521 by 3Clpro (Fig. 2*I*). Alignment the RNF20 sequences revealed that this cleavage site is well conserved across species, including human and bat (Fig. 2*J*).

### RNF20/RFN40 Complex Inhibits SARS-CoV-2 Replication.

**Fig. 3.**
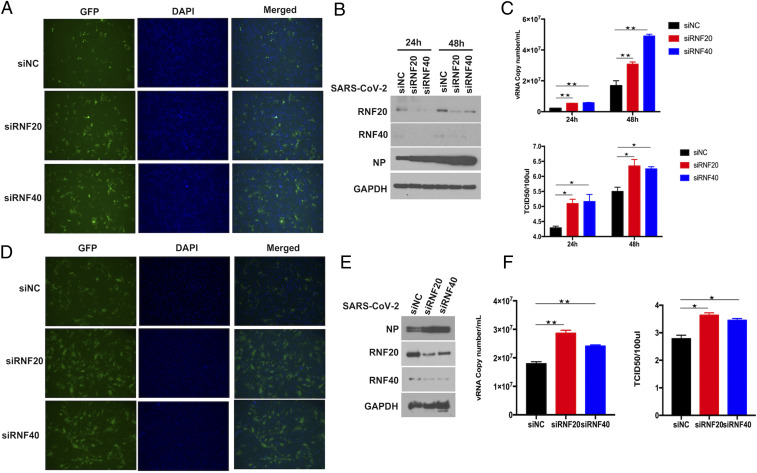
RNF20/RNF40 complex inhibits SARS-CoV-2 replication. (*A*) Microscopy images of Huh7 cells transfected with Scramble siRNA (siNC) and siRNA against RNF20 or RNF40. At 48 h posttransfection, cells were challenged with SARS-CoV-2 GFP reporter virus (0.01 MOI) for 24 h to monitor SARS-CoV-2 replication. (*B*) The siRNA targeting RNF20 or RNF40 was transfected in Huh7 cells, which were infected at 48 h posttransfection with SARS-CoV-2 GFP reporter virus at an MOI of 0.01 and incubated for additional 24 h or 48 h. SARS-CoV-2 nucleocapsid (N) protein expression level was determined by Western blot. (*C*) The levels of SARS-CoV-2 genome copy in the supernatant were determined by real-time PCR assay. The supernatants were also harvested for viral titer measurement by TCID_50_ assay performed in Vero-E6 cells. (*D*) ACE2-HeLa cells were transfected with siRNA against the indicated genes for 48 h and then infected with SARS-CoV-2 GFP reporter virus at an MOI of 0.01 and incubated for 24 h. The GFP protein was observed by fluorescence microscopy. (*E*) The indicated siRNAs were transfected in ACE2-HeLa cells, which were infected 48 h later with SARS-CoV-2 GFP reporter virus at an MOI of 0.01 and incubated for an additional 24 h. SARS-CoV-2 nucleocapsid (N) protein expression level was determined by Western blot. (*F*) The virus titers were evaluated by TCID_50_ assay. Virus RNA copy numbers in the supernatant were measured by real-time PCR. Magnification is 10X for all fluorescene microscopy images. Results are expressed as the mean ± SD (error bar) of three independent experiments; asterisks in *C* and *F* represent statistical significance based on two-tailed unpaired Student’s *t* test (*P < 0.05, **P < 0.01).

To explore the significance of RNF20 cleavage by 3Clpro, we performed small interfering RNA (siRNA)-mediated knockdown experiments to determine the effects of RNF20 in SARS-CoV-2 replication. Compared with the negative control siRNA, pooled siRNAs reduced RNF20 protein expression in Huh7 and ACE2-HeLa cells (Fig. 3 *B* and *E*). To analyze the effect of the RNF20 knockdown on SARS-CoV-2 replication, siRNA-treated cells were challenged with SARS-CoV-2 at the indicated time. As shown in Fig. 3 *B* and *E*, SARS-CoV-2 nucleocapsid (N) was increased in response to the silencing of RNF20, compared to the cells transfected with Scramble siRNA. Virus titer assays (50% tissue culture infectious dose [TCID_50_]) and viral genome copies also indicated that knockdown of RNF20 significantly promotes viral replication (Fig. 3 *C* and *F*). Immunofluorescence analysis of GFP reporter virus showed that the number of florescent foci was higher in SARS-CoV-2− infected RNF20 knockdown cells than in control cells (Fig. 3 *A* and *D*).

Since RNF20 forms the heterodimer with RNF40 to mutually stabilize each other, which is required for their E3 ligase activity, reduced expression of RNF40 protein was also observed under the overexpression of 3Clpro, along with the cleavage of RNF20 (*SI Appendix*, Fig. S2). Knockdown of RNF40 also led to the reduction of RNF20 protein level, which is consistent with the above findings that RNF20 silencing by siRNAs also interferes the expression of RNF40. Next, in the same experimental setting, virus titers and viral genome copies in the RNF40 siRNA-treated cells were significantly higher than that of the Scramble siRNA control (Fig. 3 *C* and *F*). Consistent with RNF20, RNF40 knockdown also gave rise to a better replication of GFP reporter virus, as evidenced by an increased level of green fluorescent protein when silencing RNF40 (Fig. 3 *A* and *D*). We also observed similar results when analysis of viral N expression was performed by immunoblotting (Fig. 3 *B* and *E*). Furthermore, we also generated RNF20- or RNF40-deficient Huh7 cells using CRISPR-Cas9. As shown in *SI Appendix*, Fig. S3, viral titer, viral genome copies, and fluorescent GFP protein were analyzed. The results show that both RNF20 and RNF40 could inhibit SARS-CoV-2 replication, which is consistent with the above results in RNF20/RNF40 knockdown Huh7 and ACE2-HeLa cells.

To further confirm the antiviral effects of RNF20/RNF40 complex in cells derived from lung that is the primary target by SARS-CoV-2, we established stable clones of A549 cells overexpressing human ACE2 and then evaluated SARS-CoV-2 replication. The results reveal that knockdown of either RNF20 or RNF40 can obviously promote viral N expression, which was consistent with the above results in Huh7 and ACE2-HeLa cells (*SI Appendix*, Fig. S4*B*). As shown in *SI Appendix*, Fig. S4, siRNA-mediated knockdown of RNF20 or RNF40 promoted the replication of SARS-CoV-2. Collectively, these results indicate that RNF20/RNF40 complex is a host restrict factor against SARS-CoV-2.

### SREBP1 Promotes SARS-CoV-2 Replication.

**Fig. 4.**
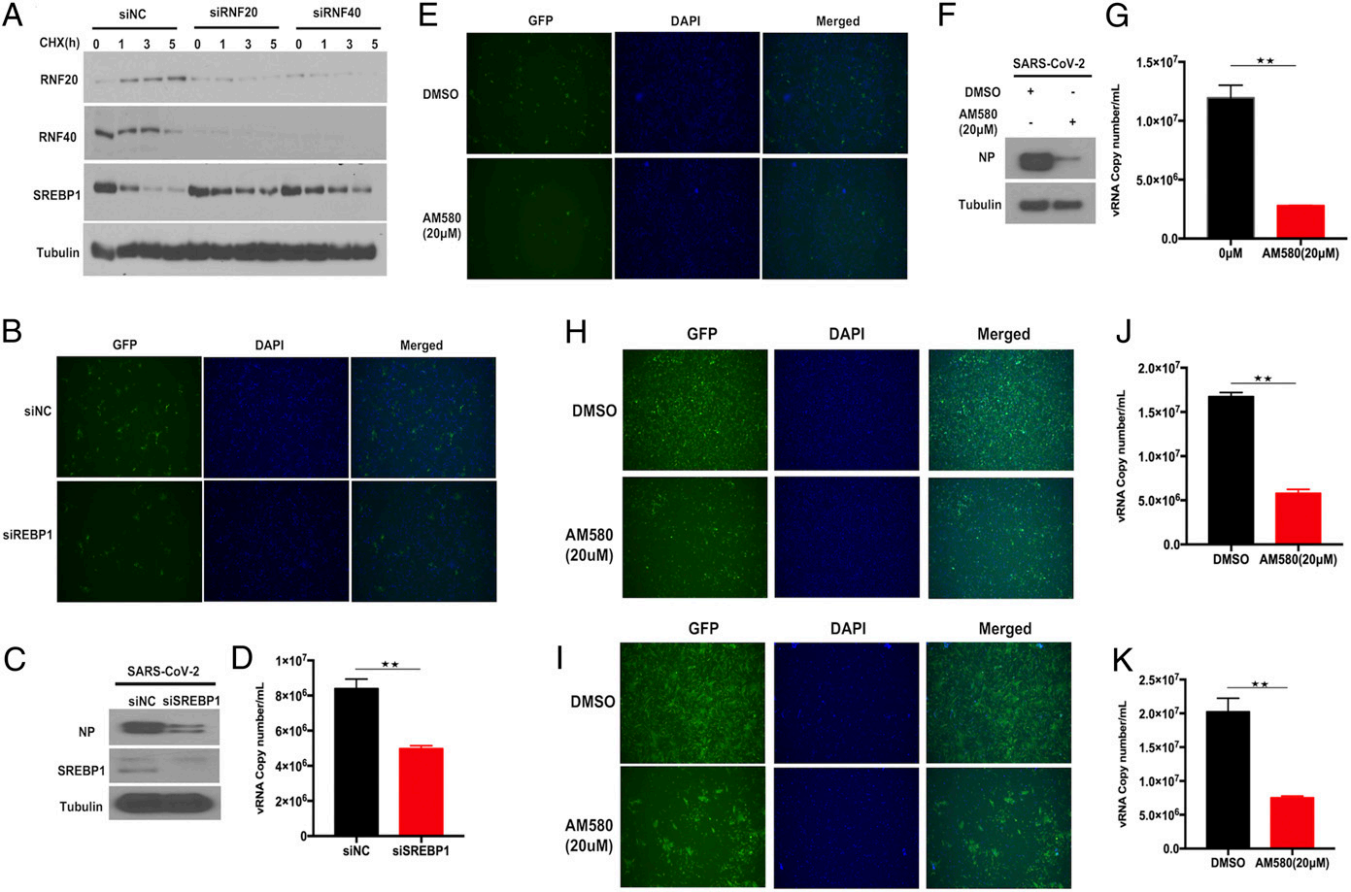
SREBP1 is required for SARS-CoV-2 replication. (*A*) The siRNA against RNF20 or RNF40, and Scramble siRNA, were introduced into Huh7 followed by treatment by cycloheximide (100 μg/mL) for 1, 3, and 5 h. Immunoblotting assay was performed and probed with antibodies against RNF20, RNF40, and SREBP1. Tubulin was used as a loading control. Huh7 cells were transfected with Scramble siRNA or siRNA against SREBP1 for 48 h, and then infected with SARS-CoV-2 GFP reporter virus at an MOI of 0.01 and incubated for 24 h. Fluorescence microscopy images of infected Huh7 cells (*B*), viral nucleocapsid (N) protein expression (*C*), and viral RNA copy numbers in the culture (*D*) were analyzed. Huh7 (*E*–*G*), Vero-E6 (*H* and *J*), and ACE2-HeLa (*I* and *K*) cells were incubated with SARS-CoV-2 GFP reporter virus at an MOI of 0.01 for 1 h, and then treated with DMSO or AM580 (20 µm) for an additional 24 h. Fluorescence microscopy analysis (*E*, *H*, and *I*), viral nucleocapsid (N) protein expression (*F*), and viral RNA copy numbers in the culture (*G*, *J*, and *K*) were performed to determine the virus replication by Western blotting or real-time PCR. Magnification is 10X for all fluorescene microscopy images. Results are expressed as the mean ± SD (error bar) of three independent experiments; asterisks in *D*, *G*, and *J* represent statistical significance based on two-tailed unpaired Student’s *t* test (**P < 0.01).

We next determined how RNF20/RNF40 complex restricts SARS-CoV-2 replication. As an E3 ligase, RNF20 plays a critical role in the regulation of lipid metabolism by modulating the transcriptional activity and protein stability of SREBP1. Thus, we hypothesized that RNF20/RNF40 complex inhibits SARS-CoV-2 replication by modulating the protein level of SREBP1. To test this hypothesis, we treated Huh7 cells with siRNA to knock down RNF20 or RNF40, followed by treatment with protein synthesis inhibitor cycloheximide for the indicated times. Consistent with the results from previous reports, the half-life of SREBP1 was prolonged in the absence of RNF20. Moreover, silencing of RNF40 in Huh7 cells also delayed endogenous SREBP1 turnover, indicating that RNF20/RNF40 complex is required for the negative regulation of SREBP1 (Fig. 4*A*).

To further study the role of SREBP1 in SARS-CoV-2 replication, Huh7 cells were transiently knocked down for SREBP1 using an siRNA approach. The fluorescence analysis revealed an impaired replication ability of SARS-CoV-2 in SREBP1 silencing Huh7 cells (Fig. 4*B*). Moreover, as observed in the SREBP1 knockdown cells, viral protein expression and RNA copies were diminished, indicating that SREBP1 is required for SARS-CoV-2 replication in Huh7 cells (Fig. 4 *C* and *D*). To further confirm our observations that SREBP1 facilitates SARS-CoV-2 replication, we treated Huh7 cells with AM580, a small-molecule inhibitor which disrupted n-SREBP1 and SRE binding, specifically via impairing the SRE-recognition functionality of n-SREBP1. Consistent with the above results in SREBP1 knockdown Huh7 cells, AM580 treatment impeded SARS-CoV-2 replication, as demonstrated by a decrease in viral protein expression and viral RNA copies, as well as fluorescence GFP (Fig. 4 *E–G*). Moreover, we also evaluated the antiviral effects of AM580 in Vero-E6 (Fig. 4 *H* and *J*) and ACE2-HeLa (Fig. 4 *I* and *K*) cells. The results show that AM580 could significantly inhibit SARS-CoV-2 replication in different cell lines. Collectively, these results indicate that SREBP1 is essential for SARS-CoV-2 replication, and AM580 would be a potential inhibitor against SARS-CoV-2.

## Discussion

In the competition between host and viruses, type I IFN provides the first line of defense against invading pathogens through driving the transcription of hundreds of ISGs which exert numerous antiviral effector functions (10, 13). To cope with theses antiviral effects of ISGs, viruses have evolved elaborate strategies to evade IFN antiviral response by inhibiting ISG induction or by hijacking specific IFN-induced antiviral proteins to promote their replication. To our knowledge, some proteins encoded by SARS-CoV-1, including nsp1, papain-like protease (PLpro), nsp7, nsp15, ORF3b, M, ORF6, and N proteins, have been documented to hijack IFN signaling to dampen ISGs production (14–17). SARS-CoV-2 infection stimulates substantial but delayed IFN production, suggesting that SARS-CoV-2 infection attenuates host antiviral response (18). In this regard, multiple SARS-CoV-2 proteins have been identified to diminish ISG expression indirectly through impairing IFN synthesis and Janus kinase−signal transducer and activator of transcription (JAK-STAT) signaling (18–20). In the current study, we reveal a potential mechanism by which SARS-CoV-2 main protease, 3Clpro, directly targets RNF20 for cleavage to escape RNF20/RNF40-mediated antiviral response against SARS-CoV-2 replication.

SARS-CoV-2 encoded PLpro has been documented to suppress IFN-mediated antiviral response through deubiquitinating and de-ISGylating activities (21–23). However, the role of 3CLpro in disrupting host antiviral immunity is largely unknown due to the covered host targets. Recently, 3CLpro was reported to inhibit both IFN production and JAK-STAT signaling to antagonize innate antiviral immunity, thus enhancing viral replication (24). Interestingly, the enzymatic activity might be necessary for 3CLpro to suppress JAK-STAT signaling, but the particular target of 3CLpro is still unclear. The histone 2B (H2B) catalyzed by the hBre1/RNF20 complex is necessary for activation of the cellular ISG expression program in response to viruses. To establish effective infection, E1A protein encoded by human adenovirus dissociates the hBre1/RNF20 complex to block IFN-induced H2B ubiquitination and associated ISG expression (25). For coronavirus, we revealed that 3Clpro can cleave and destabilize RNF20/RNF40 complex, which may provide a potential mechanism by which 3Clpro restrains the IFN-mediated response. Multiple studies have shown that the viral accessory protein Orf6 of SARS-CoV and SARS-CoV-2 is able to inhibit STAT1 nuclear translocation to block IFN signaling (19, 20). The established intraviral and viral−host interactomes reveal that ORF6 would associate with the host ubiquitin pathway, including RNF20, RNF40, RFWD3, and MyCBP1 (E3 ligase) (26), suggesting the potential modulation of the RNF20/RNF40 complex by ORF6 and 3CLpro.

We have further provided evidence that RNF20 cleavage by 3Clpro prevents RNF20/RNF40-mediated degradation of SREBP1 which is essential for SARS-CoV-2 replication. SREBPs are a family of endoplasmic reticulum membrane-bound transcription factors that regulate the synthesis of cholesterol and fatty acids by controlling the expression of a range of enzymes required for endogenous cholesterol, fatty acid, triacylglycerol, and phospholipid synthesis (27). The importance of SREBPs has been implicated in the life cycle of diverse viruses. The 25-hydroxycholesterol (25HC), a cholesterol metabolite inhibiting activation of SREBP1, suppresses infection of a broad range of pathogenic viruses, including influenza virus (28), Zika (29), and SARS-CoV-2 (30–32). AM580, a retinoid derivative and RAR-α agonist, is highly potent in interrupting the life cycle of diverse viruses, including MERS-CoV and influenza A virus, through blocking nuclear SREBP binding to a sterol regulatory element, thus suppressing SREBP-dependent lipogenic transactivation (33). Recently, two independent groups reported that knockdown of SREBP2 impaired infection by all coronaviruses in Huh7 cells (34, 35). Coupled with these findings, our results showed that SREBP1 is also essential for SARS-CoV-2 replication, highlight the importance of SREBPs in SARS-CoV-2 replication, and indicate targeting SREBPs for the development of effective antivirals. SARS-CoV-2 infection of human primary monocytes up-regulated SREBP-1 (36).

In summary, we identify RNF20/RNF40 complex as the proteolytic target of SARS-CoV-2 main protease 3CLpro and reveal its important role in SARS-CoV-2 replication. We demonstrate that 3CLpro rescues SREBP1 from RNF20/RNF40 complex-mediated degradation, which is essential for viral replication. Our studies suggest that 3CLpro inhibitors may suppress SARS-CoV-2 replication by blocking cleavage of not only viral polyproteins but also host antiviral factors such as RNF20. Future efforts will focus on dissecting the interplay between SARS-CoV-2 and host lipid metabolism, which might further contribute to the development of novel therapeutics against COVID-19.

## Materials and Methods

### Viruses and Cell Lines.

SARS-CoV-2 virus was kindly provided by NIH, and fluorescently tagged SARS-CoV-2 was kindly provided by Pei-yong Shi at University of Texas Medical Branch in Galveston, TX. Viruses were propagated in Vero-E6 cells and titrated by TCID_50_. Cells were infected with SARS-CoV-2 at the indicated multiplicity of infection (MOI) in Opti-MEM for 1 h and then cultured with fresh medium supplemented with 2% fetal bovine serum (FBS). All experiments with the SARS-CoV-2 virus were performed in the BSL-3 laboratory of University of California, Los Angeles (UCLA). Huh7 and A549 cells were purchased from ATCC and cultured in Dulbecco’s modified Eagle’s medium (DMEM; ThermoFisher) supplemented with 10% FBS (HyClone), and 1% penicillin-streptomycin (Gibco) at 37 °C in a 5% CO_2_ humidified atmosphere.

### Real-Time PCR.

Huh7 and A549-hACE2 cells were infected with SARS-CoV-2 at an MOI of 0.01 for the indicated time. Then 200 μL of the supernatant culture was harvested for viral RNA extraction with the high pure viral RNA kit (Invitrogen) or virus titer evaluation. RNA was finally eluted with 30 μL of RNase-free water and used as the template for RT-PCR quantification. For qPCR analysis, the specific primers that target SARS-CoV-2 N gene were used: forward, 5′-TAA​TCA​GAC​AAG​GAA​CTG​ATT​A-3′; reverse, 5′-CGA​AGG​TGT​GAC​TTC​CAT​G-3′. RT-qPCR was performed using One Step TB Green PrimeScript RT-PCR Kit II (Takara) with the following cycling conditions: 42 °C for 5 min, 95 °C for 10 s, and 40 cycles of 95 °C for 5 s, followed by 60 °C for 30 s.

### TCID_50_ Assay.

For virus titration, SARS-CoV-2 titer in the culture was determined by TCID_50_ assay. Briefly, serial 10-fold dilutions of virus samples were prepared in 900 μL of Opti-MEM; then 100 μL of diluted viral inoculum was added to Vero-E6 cells in eight replicates in a 96-well culture plate. After 1 h of incubation, the medium was changed into DMEM with 2% FBS for an additional 3 d to 4 d in a 5% CO_2_ incubator at 37 °C. Cytopathic effect was checked under a microscope, and TCID_50_ was calculated.

### Immunoblotting.

Cells were lysed with Lysis buffer (50mM Tris-Cl pH8.0, 5 mM (ethylenedinitrilo)tetraacetic acid, 150 mM NaCl, 0.5% Nonidet P-40) plus protease inhibitor mixture. Whole cell lysates were obtained by centrifugation at 12,000 rpm for 15 min at 4 °C and then denatured with 1×LDS sample buffer at 95 °C for 10 min. For the collection of cell lysates after virus infection, 2xLDS sample buffer was added into the cell samples after removing the culture medium, and then denatured at 95 °C for 10 min. Lysates were electrophoresed in 10% polyacrylamide gels followed by immunoblotting with the indicated antibodies.

### Statistical Analysis.

Experimental data are presented as mean ± SD. All statistical analyses were performed using a two-tailed Student’s *t* test. Differences were considered statistically significant when *P* values were less than 0.05. *P < 0.05, and **P < 0.01 for all the analysis.

## Supplementary Material

Supplementary File

## Data Availability

All study data are included in the article and *SI Appendix*.
