# Supplementary material for: Protease cleavage of RNF20 facilitates coronavirus replication via stabilization of SREBP1
Source: Proc Natl Acad Sci U S A. 2021 Aug 27;118(37):e2107108118. doi: 10.1073/pnas.2107108118 (PMC8449311; doi:10.1073/pnas.2107108118)
Supplement: Supplementary File [file pnas.2107108118.sapp.pdf]

PNAS
www.pnas.org

Supplementary Information for

Protease Cleavage of RNF20 Facilitates Coronavirus Replication via Stabilization of SREBP1

Shilei Zhang[a], Jingfeng Wang[a,b,c], Genhong Cheng[a,1]

[a]Department of Microbiology, Immunology, and Molecular Genetics, University of California, Los Angeles, Los Angeles, CA 90095; [b]Institute of Systems Medicine, Chinese Academy of Medical Sciences & Peking Union Medical College, Beijing 100005, China; [c]Suzhou Institute of System Medicine, Suzhou, Jiangsu 215123, China.

Corresponding author: Genhong Cheng

Email: gcheng@mednet.ucla.edu.

This PDF file includes:

Supplementary text
Figures S1 to S5
Legends for Figures S1 to S5

Supplementary Materials and Methods

Plasmids. pCMV-3xHA-NSP5 and pCMV-3xFlag-NSP5 were generously provided by Dr. Shitao Li (Tulane University School of Medicine). pLENTI-hACE2-PURO was a gift from Raffaele De Francesco. All the predicated cleavage sequences were amplified by KOD hot start DNA polymerase (Sigma-Aldrich, USA) and subcloned into the vector pEFG-C3 by In-Fusion HD Cloning system (Takara, Japan). Full-length SLC25A22 was amplified from human cDNA and subcloned into the vector pCDNA-3xFlag. RNF20 and BIRC6 cDNA were synthesized and subcloned into the vector pReceiver-M01. Human RNF20 Gene ORF2 cDNA clone expression plasmid fused N-terminally to the pCMV3-Flag were from Sino Biological. Both N-terminal fragment (residues 1-521) and C-terminal fragment (residues 522-975) were amplified and cloned into plasmid pCDNA-3xFlag. PCR-amplified segments of NSP7 and NSP8 cDNA (were kindly provided by Dr. Vaithi Arumugaswami, UCLA) were subcloned into plasmid pGFP-C3 to generate a NSP7/NSP8 fused expression plasmid. Point mutations were introduced by PCR, using KOD hot star DNA polymerase (Sigma-Aldrich, USA). For transient expression, HEK293T cells were transfected with the indicated plasmids using the PEI method. After 24 h, cell lysates were prepared to further analysis. For siRNA transfection, siRNA pools of target gene were introduced into Huh7 or A549-hACE2 cells using Lipofectamine RNAiMAX Reagent (ThermoFisher, USA) according to the manufacturer's instruction for 48h, followed by SARS-CoV-2 infection at the 0.01 MOI for an additional 24 h or 48 h.

siRNA and antibodies. The following siRNAs targeting human genes were used: control siRNA-A is non-targeting 20-25nt siRNA designed as a negative control (sc-37007); RNF20 siRNA (sc-92753); RNF40 siRNA (sc-93054); SREBP-1 siRNA (sc-36557); Each of siRNA against RNF20, RNF40 or SREBP1 is a pool of 3 target-specific 19-25nt siRNAs designed to knock down the indicated gene expression. Resuspension of the siRNA duplex in 330 µl of RNAse-free water makes a 10µM solution in a 10µM Tris-HCl, pH 8.0, 20mM NaCl, 1mM EDTA buffered solution.

Primary antibodies used in this study were: Anti-GFP(D5.1) Rabbit mAb (Cell signaling, #2956); Anti-HA-Tag (C29F4) Rabbit mAb (Cell signaling, #3724); Monoclonal ANTI-FLAG® M2 antibody produced in mouse (Sigma, F1804); GAPDH (14C10) Rabbit mAb (Cell signaling, #2118); RNF40 (D2R2O) Rabbit mAb (Cell signaling, #12187); RNF20 Polyclonal Antibody (Proteintech, 21625-1-AP); SREBF1 Polyclonal antibody (Proteintech, 140881-1-AP); SARS-CoV-2 (COVID-19) nucleocapsid antibody (GeneTex, GTX635679); Alpha Tubulin Polyclonal antibody (Proteintech,11224-1-AP). Secondary antibodies were: Goat Anti-Rabbit IgG(H+L), Mouse/Human ads-HRP (Southernbiotech, 4050-05); Goat Anti-Mouse IgG(H+L), Human ads-HRP (Southernbiotech, 1031-05).

Inhibitors and compounds. The following Inhibitors and compounds were used: AM580 (MedChemExpress, HY-10475); MG-132, Ready Made Solution (Sigma, M7449); MLN4924 (MedChemExpress, HY-70062); GC376 (Selleck, S0475); Protease inhibitor cocktail (Sigma, 11836153001).

PEI and Lipofectamine Transfection. 1 mg/mL polyethylenimine (PEI) was used to introduce plasmids into HEK293T cells. HEK293T cells were seeded in the 12 wells plate for incubation at

37 ℃, 5% CO_2 for 20h. The plasmids were added to the 100μL Opti-MEM medium and briefly mixed. Then the exact amount of PEI (Ratio of DNA/PEI: 1μg DNA/3μL PEI) was added to the 100μL diluted plasmids, briefly mixed and kept at room temperature for 20min. The 100 μL of PEI/DNA complex was added drop-wise to the wells and the cells were incubated at 37°C for 24h. All siRNA transfections were carried out in 12 wells plates using Lipofectamine RNAiMAX Reagent (ThermoFisher, USA). For the transfection with RNAiMAX, 1μL of the siRNA duplex(10μM) and 2μL of RNAiMAX reagent was separately diluted into 50μL Opti-MEM medium and left at room temperature for 5min, and then the 50μL of siRNA duplex/Opti-MEM was added to the 50μL RNAiMAX/Opti-MEM solution, briefly mixed and kept at room temperature for additional 20min. The culture medium was replaced with 400μL Opti-MEM, and then the 100μL siRNA/RNAiMAX complexes were added drop-wise to the plates for incubation at 37°C. After 8h post-incubation, the siRNA/RNAiMAX was removed and the fresh medium with 2% FBS was added following incubation at 37°C for additional 48h.

Pseudotyped lentiviral-mediated Gene Modification. Reconstitution of human ACE2 into A549 cells were performed by pseudotyped lentiviral-mediated gene modification. HEK293T cells in 10cm dishes were transfected with the lentiviral expression plasmid carrying ACE2 (9μg) together with the pMD2.G (3μg) and pPAX2 (6μg) plasmids by using PEI. To generate RNF20 and RNF40 knock-out Huh7 cells, HEK293T cells in 10cm dishes were transfected with the lentiviral-Cas9-V2 expression plasmid carrying sgRNA against RNF20 or RNF40 (7.2μg) together with the pMD2.G (5μg) and pPAX2 (7.2μg) plasmids using PEI. At 48h post-transfection, pseudotyped viruses in the supernatant were collected and centrifuged at 1500rpm for 10min to remove cell debris. The pseudotyped viruses and were incubated with A549 cells or Huh7 cells (in 6 wells plate) in the presence of polybrene (8ug/ml), and then spin at 1500rpm for 60min at room temperature to help increase the transduction efficiency. At 8h after spin transduction, replace medium with fresh complete medium to remove polybrene. The positive cells were selected with puromycin (2μg/ml for A549 cells, 1μg/ml for Huh7 cells) at 48h post transduction. Immunoblot assay was performed to verify the indicated proteins expression.

Immunofluorescence microscopy. Cells were washed with PBS and fixed with 4% paraformaldehyde in PBS for 15 min at room temperature. Cells were washed with PBS for 3 times and then stained with 0.1 μg/ml DAPI (ThermoFisher, 62248) for 5min. Fluorescence was observed using Leica Fluorescence Microscope, and Images were captured and processed using LAS X Multi-channel acquisition software.

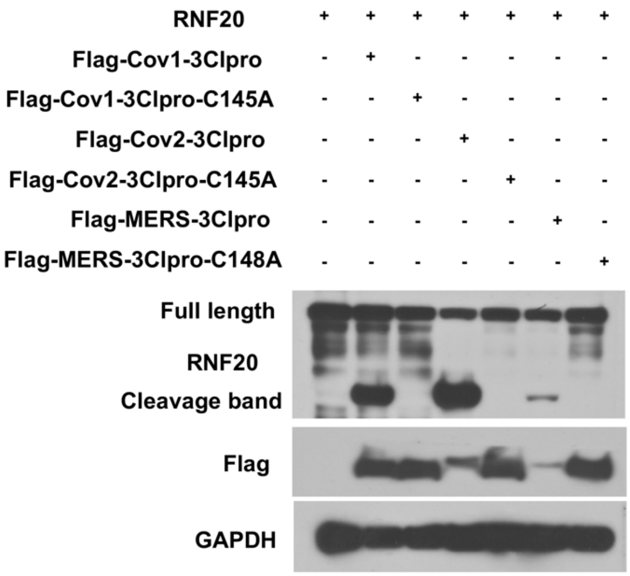

FigS1. RNF20 cleavage by 3Clpro is conserved among Coronaviruses. Plasmid expressing RNF20 (200ng) was transfected into HEK293T cells with the equivalent plasmids expressing SARS-CoV-1, SARS-CoV-2, and MERS-CoV 3Clpro and their enzymatic dead mutants. At 24h post-transfection, cell lysates were analyzed by Western blot to show the cleavage of RNF20. GAPDH was used with loading control.

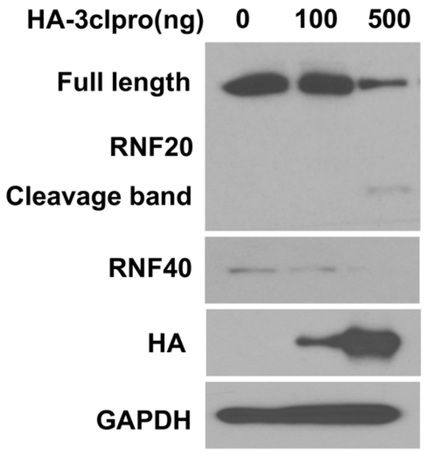

FigS2. Cleavage RNF20 by 3Clpro reduces endogenous RNF40 protein. HEK293T cells were transfected with 0, 100, or 500 ng of HA-3Clpro–expressing plasmids for 24h. Expression of endogenous RNF40 and cleavage of endogenous RNF20 were evaluated by Western blotting.

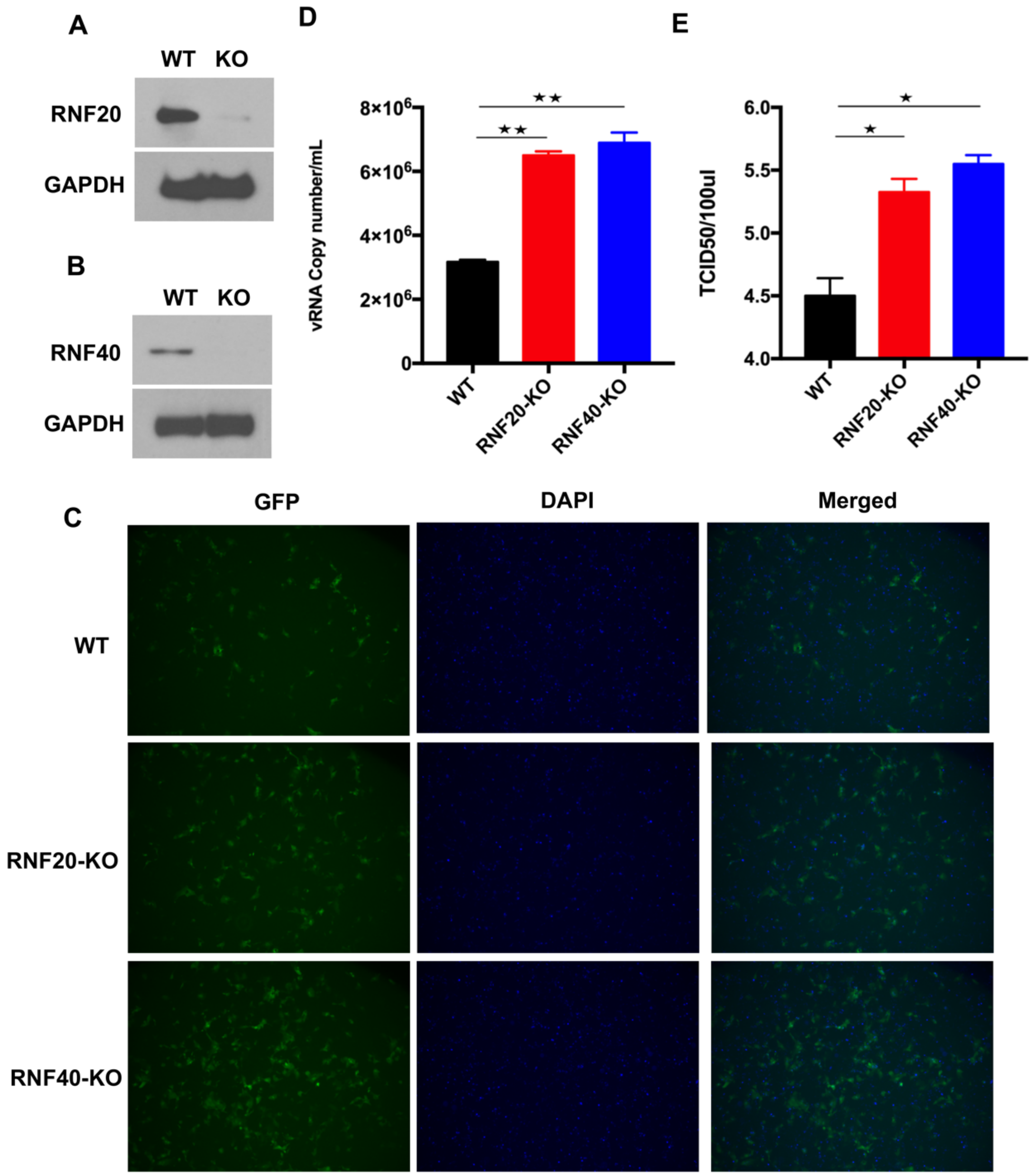

FigS3. RNF20/RNF40 deficiency promotes SARS-CoV-2 replication in Huh7 cells. (A, B) CRISPR-Cas9-mediated knockout of RNF20 or RNF40 in Huh7 cells. Immunoblotting was performed to validate the RNF20 and RNF40 protein level after CRISPR editing. **(C)** RNF20 and RNF40 deficiency Huh7 cells were infected with SARS-CoV-2 GFP reporter virus (0.01MOI) for 24h. The viral replication was observed by fluorescence microscopy. **(D)** The levels of SARS-CoV-2 genome copy in the supernatant were determined by Real-time PCR assay. **(E)**The supernatant was also harvested for viral titer measurement by TCID50 assay performed in Vero-E6 cells. Asterisks (D and E) represent statistical significance based on two-tailed unpaired Student’s t test (*P < 0.05, **P < 0.01).

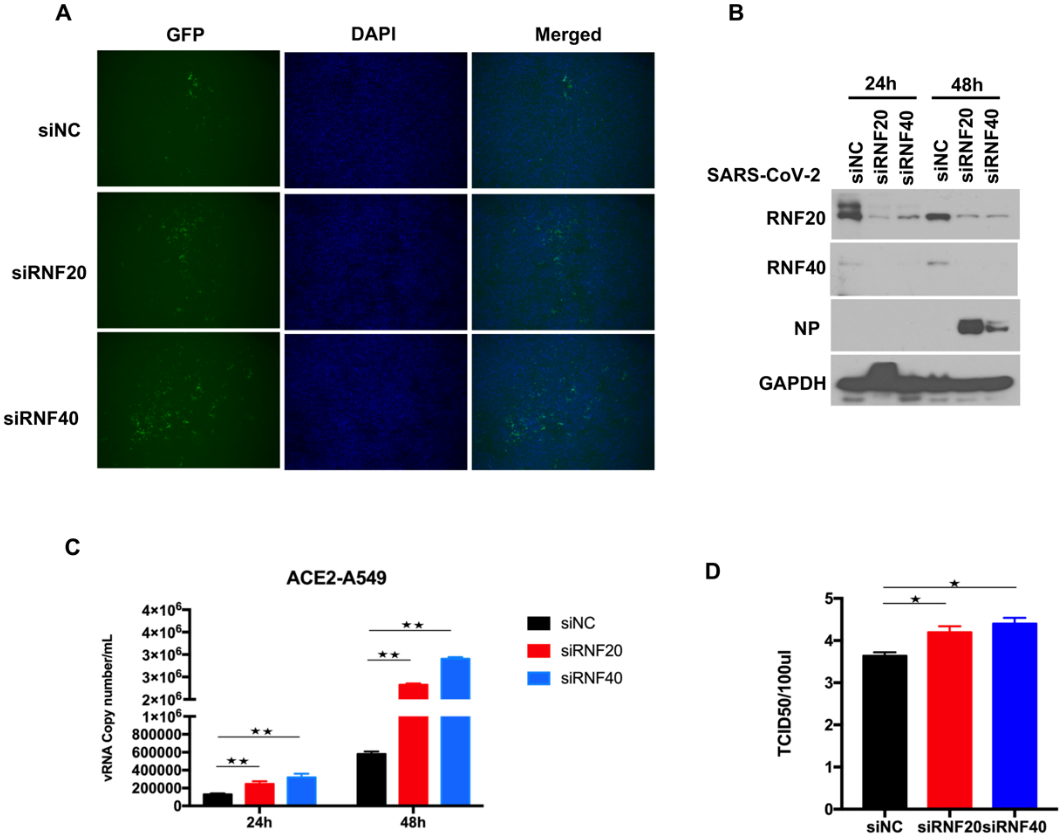

FigS4. RNF20/RNF40 complex inhibits SARS-CoV-2 replication in ACE2-A549 cells. (A) Microscopy images of ACE2-A549 cells transfected with Scramble siRNA and siRNA against RNF20 or RNF40. At 48h post-transfection, cells were challenged with SARS-CoV-2 GFP reporter virus (0.01MOI) for 24h to monitor SARS-CoV-2 replication. **(B)** siRNA targeting RNF20 or RNF40 was transfected in ACE2-A549 cells for 48h, which were then infected with SARS-CoV-2 GFP reporter virus at an MOI of 0.01 and incubated for an additional 24h or 48 h. SARS-COV-2 nucleocapsid (N) protein expression level was determined by Western blot. GAPDH was used with loading control. **(C)** The levels of SARS-CoV-2 genome copy in the supernatant were determined by Real-time PCR assay. **(D)** At 48h post-infection with SARS-CoV-2 in RNF20 or RNF40 knockdown ACE2-A549 cells, the supernatants were harvested for viral titer measurement by TCID50 assay performed in Vero-E6 cells. Asterisks (C and D) represent statistical significance based on two-tailed unpaired Student's t test (*$P < 0.05$, **$P < 0.01$).

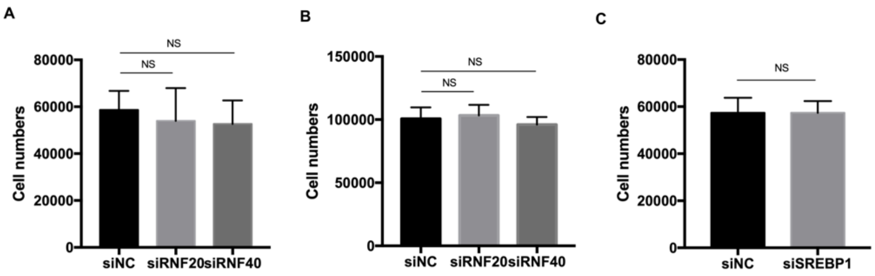

FigS5. Cell numbers counting. (A) Cell numbers in Figure3A. siRNA targeting RNF20 or RNF40 was transfected in Huh7 cells for 48h. Cells were harvested and treated with Trypan-blue solution. The numbers of viable cells were counted. **(B)** Cell numbers in Figure3D. ACE2-HeLa cells were transfected with the indicated siRNAs for 48h. The Trypan Blue dye exclusion test is used to determine the number of viable cells present in a cell suspension. **(C)** Cell numbers in Figure4B. At 48h post-transfection with the siRNAs against SREBP1 in Huh7 cells, the cell suspension was prepared for number counting using Trypan-blue staining. *NS* represents statistical significance based on two-tailed unpaired Student's t test (*P < 0.05, **P < 0.01, *NS*>0.05).
